# Building personalized treatment plans for early-stage colorectal cancer patients

**DOI:** 10.18632/oncotarget.14638

**Published:** 2017-01-13

**Authors:** Hung-Hsin Lin, Nien-Chih Wei, Teh-Ying Chou, Chun-Chi Lin, Yuan-Tsu Lan, Shin-Ching Chang, Huann-Sheng Wang, Shung-Haur Yang, Wei-Shone Chen, Tzu-Chen Lin, Jen-Kou Lin, Jeng-Kai Jiang

**Affiliations:** ^1^ Division of Colon and Rectal Surgery, Department of Surgery, Taipei Veterans General Hospital, Taiwan; ^2^ Department of Surgery, School of Medicine, National Yang-Ming University, Taiwan; ^3^ Auspex Diagnostics, Taiwan; ^4^ Division of Molecular Pathology, Department of Pathology and Laboratory Medicine, Taipei Veterans General Hospital, Taipei, Taiwan; ^5^ Institute of Clinical Medicine, School of Medicine, National Yang-Ming University, Taipei, Taiwan

**Keywords:** recurrence, drug efficacy, microarray, colorectal cancer, personalized treatment

## Abstract

We developed a series of models to predict the likelihood of recurrence and the response to chemotherapy for the personalized treatment of stage I and II colorectal cancer patients. A recurrence prediction model was developed from 235 stage I/II patients. The model successfully distinguished between high-risk and low-risk groups, with a hazard ratio of recurrence of 4.66 (*p* < 0.0001). More importantly, the model was accurate for both stage I (hazard ratio = 5.87, *p* = 0.0006) and stage II (hazard ratio = 4.30, *p* < 0.0001) disease. This model performed much better than the Oncotype and ColoPrint commercial services in identifying patients at high risk for stage II recurrence. And unlike the commercial services, the robust model included recurrence prediction for stage I patients. As stage I/II CRC patients usually do not receive chemotherapy, we generated chemotherapy efficacy prediction models with data from 358 stage III patients. The predictions were highly accurate: the hazard ratio of recurrence for responders vs. non-responders was 4.13 for those treated with FOLFOX (*p* < 0.0001), and 3.16 (*p* = 0.0012) for those treated with fluorouracil. We have thus created a prognostic model that accurately identifies patients at high risk for recurrence, and the first accurate chemotherapy efficacy prediction model for individual patients. In the future, complete personalized treatment plans for stage I/II patients may be developed if the drug prediction models generated from stage III patients are verified to be effective for stage I and II patients in prospective studies.

## INTRODUCTION

Colorectal cancer (CRC) is one of the leading causes of cancer-related mortality worldwide. Currently, the prognosis for CRC patients is determined by pathological features and the stage of the tumor at diagnosis. Patients with American Joint Committee on Cancer defined stage I and II disease have up to a 30% chance of recurrence after surgical resection, whereas patients with stage III disease have a 50-60% chance of recurrence within five years [1–3].

For early-stage cancer patients who receive curative resection, the identification of their risk for recurrence (and thus the potential benefit of adjuvant therapy) could improve long-term outcomes. Both prognostic models that identify high-risk patients and chemotherapy efficacy prediction models that determine the efficacy of adjuvant treatments are necessary for building personalized treatment plans.

Currently, adjuvant therapy is standard care for patients with stage III CRC with survival benefit [4]. The role of adjuvant chemotherapy in stage I patients remains controversial, as most patients have good prognoses [5] and the few high-risk patients are difficult to identify. Much effort has been made to identify high-risk stage II patients who might benefit from adjuvant therapy. The National Comprehensive Cancer Network guideline has identified several factors that predict poor prognosis, including emergency presentation (tumor obstruction, perforation), an inadequate number of assessed lymph nodes (< 12), T4 tumors, poor histological differentiation, lymphovascular invasion, perineural invasion, and the presence of positive resection margins. However, these clinicopathologic factors alone have not been effective in identifying high-risk stage II patients. High-risk patients identified by the National Comprehensive Cancer Network guideline received no benefit from adjuvant chemotherapeutic treatment [6–8], and low-risk patients incurred the additional risk of worse disease-free survival (DFS) [6]. Some studies on clinicopathologic variables have indicated that only a subset of factors, such as T4 status and serum carcinoembryonic antigen (CEA) levels, effectively identified high-risk patients [9–11]. Adding DNA mismatch repair status to clinicopathologic variables has also been reported to improve prognostic predictions for stage II patients [12, 13].

Due to the limitations of clinicopathologic variables for prognostic prediction, genomic information has been increasingly used to determine the risk of recurrence [14–29]. Nevertheless, several comprehensive reviews have concluded that genomic predictions are only “marginally” better than clinicopathologic variables in predicting the prognosis for stage II CRC patients [12, 17, 30–32].

Chemotherapy efficacy prediction analysis is an important complement to prognostic analysis. For stage I and II high-risk CRC patients identified by prognostic analysis, chemotherapy efficacy prediction analysis can inform the selection of an effective adjuvant treatment [12]. Among stage III patients, about 40% will experience recurrence even after adjuvant treatment [33]; thus, chemotherapy efficacy predictions would help stage III patients weigh the benefits of treatment against potential adverse effects.

For early-stage colon cancer patients, the most commonly used adjuvant treatments are 5FU (5-fluorouracil and leucovorin) and FOLFOX (5-fluorouracil, leucovorin, and oxaliplatin). Many population-based studies have been performed [33–37] to compare the efficacy of 5FU and FOLFOX in patients of different ages and disease stages, examine the adverse effects of treatment in elderly or stage II patients, and evaluate the adverse effects [38] and cost effectiveness of adding Oxaliplatin [39, 40]. Many of these reports were based on two large clinical trials (MOSAIC and NSABP C-07).

On the other hand, there have been limited results from genomic studies addressing the issues raised by the population-based studies [41–45]. In almost all 5FU and FOLFOX efficacy studies, individual marker genes have been used to predict the effectiveness of the drug regimens. The goal of these studies was to validate the target gene functions, rather than to identify global gene signatures that would best distinguish treatment responders from non-responders. Microsatellite instability (MSI) is a successful marker that has been confirmed as a prognostic indicator, but not as a chemotherapy efficacy prediction indicator [13, 4]. Studies combining markers for the prediction of 5FU efficacy are still in their early stages [41, 42]; therefore, no markers are currently being used to predict drug efficacy in the clinic [46, 47].

In addition, many studies have been designed for stage IV patients, such that drug efficacy has been defined by a reduction in tumor size. However, in early-stage CRC patients, efficacy is better defined by recurrence after curative resection. In this study, recurrence will be used for the determination of efficacy for stage I and II patients.

In most published prognostic CRC studies, stage II patients have been evaluated; stage III patients have been included in some studies, but stage I patients have only been assessed in one publication, with a very small sample size of 15 [26]. The focus on stage II cancer patients is understandable, as such patients would be more likely to benefit from treatment, and would be easier to study than stage I patients. Prediction models are often effective for patients at one stage, but not for another. Predicting recurrence for early-stage patients requires excellent sensitivity so that rare recurrence cases can be detected, whereas predicting recurrence for later stage patients requires better specificity so that patients do not undergo unnecessary adjuvant treatment. Since adjuvant therapy is already recommended for patients with stage III CRC, the goal of the present recurrence study was to develop a robust prognostic model for stage I and II patients. We also developed the first chemotherapeutic efficacy prediction models for 5FU and FOLFOX. The goal was not only to predict the efficacy of 5FU and FOLFOX independently, but also to generate consistent predictions from these two models so that a rational choice could be made between the two treatment options.

## RESULTS

### Recurrence prediction

### Clinical data

One hundred fifty-seven samples were used as a training set to generate a prognostic prediction model. An additional 78 samples were used as a blind test set to validate the prediction model. Patients with positive resection margins were excluded from this study. Only four of the total 235 stage I/II CRC patients had T4 status. Table 1 displays the clinicopathologic features of the patients in the training and testing sets. The follow-up times and disease–free intervals did not differ significantly between the training and testing sets.

**Table 1:** Demographics of patients in the recurrence study

Variable	Total		Training Set		Test Set		*p* value
	*n* = 235	%	*n* = 157	%	*n* = 78	%	
Age (Mean ± SD)	67.8 ± 11.7		68.7 ± 11.2		66.0 ± 12.5		0.516
Follow-up (Months± SD)	65.1 ± 23.6		64.5 ± 24.3		66.5 ± 22.0		0.718
Gender							
Male	145	61.7	100	63.7	45	57.7	
Female	90	38.3	57	36.3	33	42.3	0.373
Location							0.561
Right colon	68	28.9	47	29.9	21	26.9	
Left colon	106	45.1	67	42.7	39	50.0	
Rectum	61	26.0	43	27.4	18	23.1	
Preoperative CEA level							0.325
< 5 ng/mL	160	68.1	104	66.2	56	71.8	
> 5 ng/mL	64	27.2	47	29.9	17	21.8	
NA	11	4.7	6	3.9	5	6.4	
Stage							0.984
I	39	16.6	26	16.6	13	16.7	
II	196	83.4	131	83.4	65	83.3	
Emergent operation							0.640
No	225	95.7	151	96.2	74	94.9	
Yes	10	4.3	6	3.8	4	5.1	
Mucinous component (> 50%)							0.581
No	228	97.0	153	97.5	75	96.2	
Yes	7	3.0	4	2.5	3	3.8	
Lymphovascular invasion							0.849
No	222	94.5	148	94.3	74	94.9	
Yes	13	5.5	9	5.7	4	5.1	
Perineural invasion							0.994
No	229	97.4	153	97.5	76	97.4	
Yes	6	2.6	4	2.5	2	2.6	
Grade of differentiation							0.726
Well/moderate	221	98.3	154	98.0	77	98.7	
Poor/undifferentiated	4	1.7	3	2.0	1	1.3	
Lymph nodes harvested							0.456
≥ 12	176	74.9	120	76.4	56	71.8	
< 12	59	25.1	37	23.6	22	28.2	
Adjuvant chemotherapy*							0.894
Yes	25	10.6	17	10.8	8	10.3	
No	210	89.4	140	89.2	70	89.7	
Recurrence							0.956
Yes	97	41.3	65	41.4	32	41.0	
No	138	58.7	92	58.6	46	59.0	

Abbreviations: CEA, carcinoembryonic antigen.

*Only patients who had recurrence despite adjuvant chemotherapy were included in the study. No patients received neoadjuvant therapy.

Univariate analyses of all training and test samples revealed that recurrence was associated with pre-operative CEA values, emergent operations, mucinous components, lymphovascular invasion, and adjuvant chemotherapy. Only preoperative CEA values and adjuvant chemotherapy were significant risk factors in multivariate analyses (Supplementary Table 1).

### Training set results

**Figure 1:**
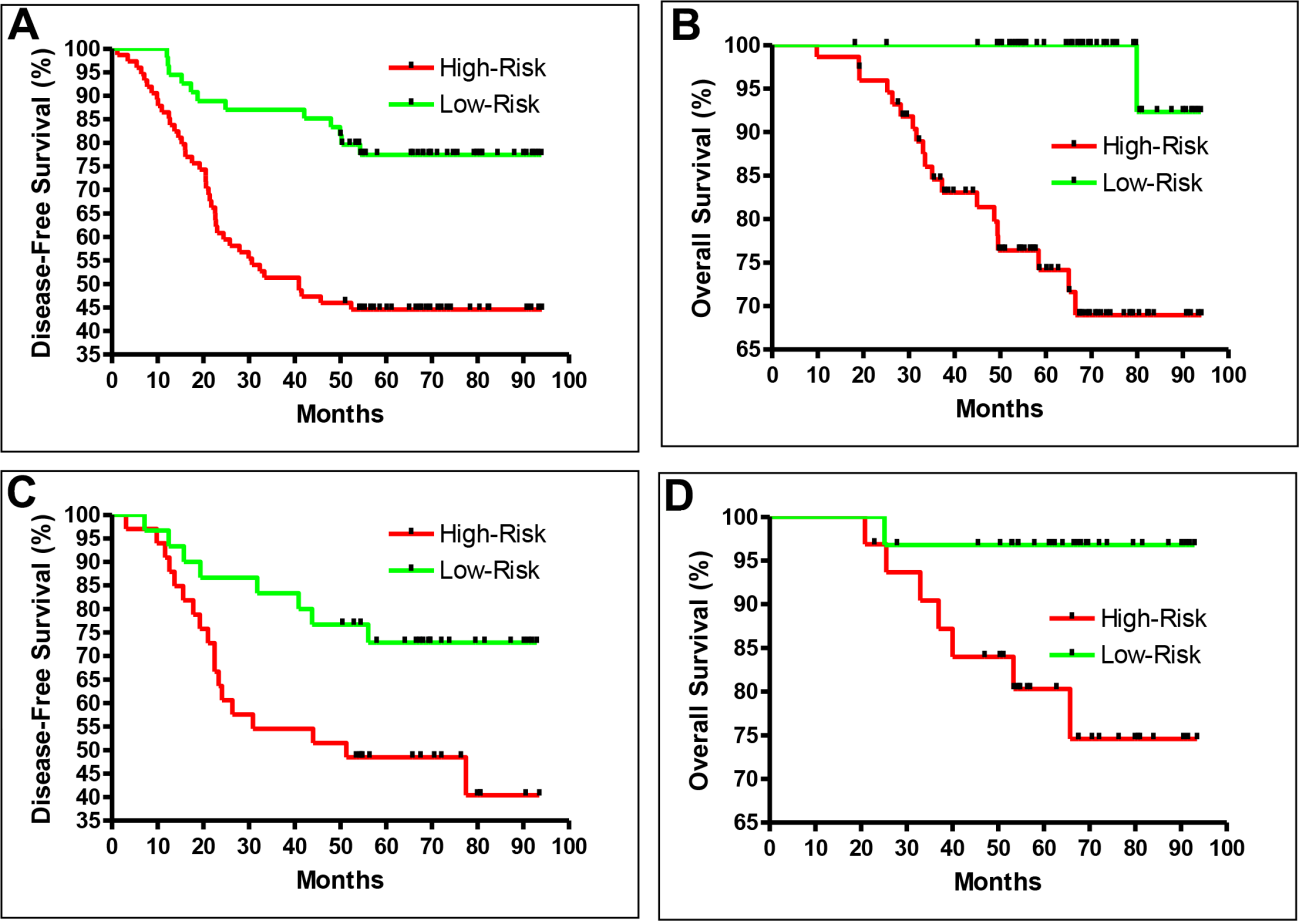
Recurrence prediction separates patient into high-risk and low-risk groups Disease-free (**A**) and overall survival (**B**) of the training samples. Disease-free (**C**) and overall survival (**D**) of the test samples. The training (A and B) and blind testing (C and D) performed similarly.

The training set consisted of 157 samples from 64 recurrent CRC patients and 93 non-recurrent patients. During the training, samples were randomly divided into two groups: 150 samples were used to generate the prediction model, and seven samples were used to test the performance of the model generated from the 150 samples by the k-nearest neighbor (KNN) method, as described in the Materials and Methods. Although the recurrence information of the seven test samples was known, the computer program was carefully designed to avoid using the recurrence information to generate the model. The training model was able to separate recurrent from non-recurrent patients effectively. The hazard ratio (HR) of recurrence in the high-risk group vs. the low-risk group was 2.90 (95% CI: 1.69 to 4.98 *P* = 0.0001; Figure 1A). In addition, in terms of overall survival, the HR in the high-risk group vs. the low-risk group was 5.61 (95% CI: 2.33 to 13.54, *P* = 0.0001; Figure 1B).

### Blind validation results

To confirm the performance of the prediction model, we used 78 additional samples (from 33 recurrent and 45 non-recurrent CRC patients) as a true blind test in which the recurrence status was not known at the prediction. The HR of recurrence in the high-risk group vs. the low-risk group in this blind test set was 2.44 (95% CI: 1.13 to 5.29, *P* = 0.0235; Figure 1C). In terms of overall survival, the HR in the high-risk group vs. the low-risk group was 4.68 (95% CI: 1.17 to 18.70, *P* = 0.0293; Figure 1D). These results confirmed the effectiveness of the recurrence prediction model.

### Final recurrence prediction results

**Figure 2:**
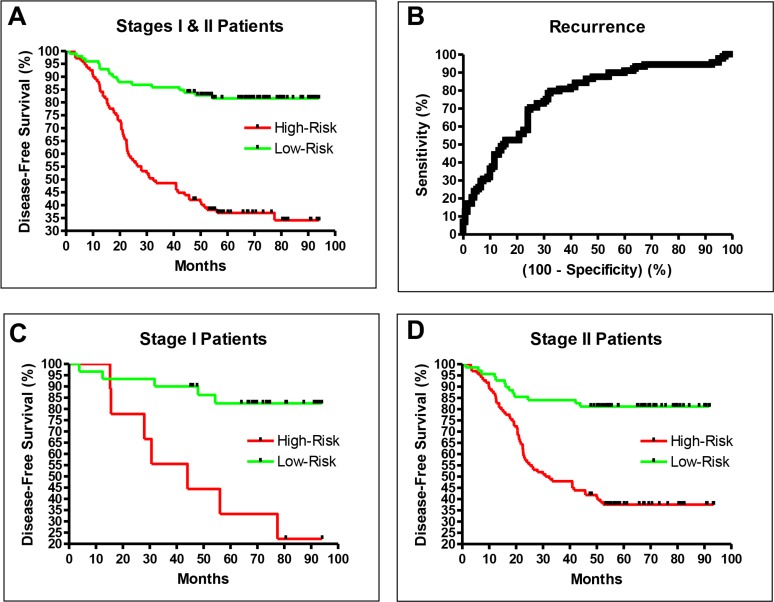
The final performance of the recurrence prediction model including all stage I and II samples is shown in (**A**) and (**B**). The performances with separated stage I and II samples are shown in (**C**) and (**D**). Patients in stages I and II had similar long-term DFS rates.

The consistency of the validation and training results demonstrated that the prediction model was robust and unbiased. To better characterize the recurrence prediction model, we studied all 235 samples (the original 157 training samples and the 78 testing samples) together, and used a leave-five-out method to characterize the final prediction performance. The HR of recurrence in the high-risk group vs. the low-risk group was 4.66 (95% CI: 2.66 to 6.25, *P* < 0.0001; Figure 2A), and the area under the curve for the Receiver Operating Characteristic curve of this prediction model was 0.77 (*P* < 0.0001; Figure 2B), with a sensitivity of 0.80 and a specificity of 0.68 when the default cutoff was used (Table 2).

**Table 2:** Performance of All three prediction models at default cutoff

Model	Sensitivity	Specificity	Accuracy	AUC	HR	HR Range	*P*-Value
Recurrence	0.80	0.68	0.73	0.77	4.66	2.69 to 6.27	< 0.0001
5FU	0.76	0.59	0.70	0.67	3.16	1.69 to 8.23	= 0.0012
FOLFOX	0.88	0.47	0.72	0.68	4.13	4.07 to 14.90	< 0.0001

Abbreviations:

HR: Hazard Ratio of High-Risk vs Low-Risk Recurrence.

HR Range: HR 95% Confidence Interval Range.

AUC: Area Under the ROC Curve.

The performance reported above was based on a 235-sample set that included both stage I and stage II CRC patients. To evaluate the prediction performance for stage I or stage II patients separately, we segregated and re-examined the samples. The HR of recurrence in stage I patients was 5.87 (95% CI: 2.99 to 53.51, *P* = 0.0006; Figure 2C), and in stage II patients was 4.30 (95% CI: 2.15 to 5.39, *P* < 0.0001; Figure 2D). Figure 2 displays the similar long-term recurrence rates for stage I and II patients, demonstrating the robustness of the prediction model.

This recurrence prediction model also performed well for all three types of CRC. The HR of recurrence was 6.81 (95% CI: 2.34 to 10.78, *P* < 0.0001) in patients with right-sided colon cancer, 4.51 (95% CI: 1.97 to 7.81, *P* < 0.0001) in patients with left-sided colon cancer, and 3.27 (95% CI: 1.43 to 6.79, *P* = 0.0042) in patients with rectal cancer.

Currently, there are two commercial services for stage II colon cancer recurrence prediction. ColoPrint obtained a five-year DFS difference of about 14% and a HR of recurrence of 2.65 between high-risk and low-risk patients using fresh frozen tissue [15]. Oncotype determined a HR of 1.43 for a recurrence score difference of 25 using formalin-fixed, paraffin-embedded (FFPE) tissue; the maximum three-year DFS difference was 14% between patients with the highest and lowest recurrence scores [14]. The actual DFS difference will be lower, and depends on the cutoff between high-risk and low-risk patients. In contrast, in this study, much better results were achieved with FFPE tissue. The HR of recurrence in high-risk patients vs. low-risk patients was 4.66 (Table 1), the five-year DFS difference between high-risk and low-risk patients was 46%, and the three-year DFS difference was 40% (Figure 2A). Thus, the DFS difference in this study was about three times greater than those of the previous studies.

### Biomarker comparison among different studies

We compared the genes selected for our recurrence prediction model (Supplementary Table 3) with those selected by the two commercial services, Oncotype and ColoPrint. While Oncotype used seven genes and ColoPrint used 18 genes (listed in the Materials and Methods section), we used 120 genes to generate our recurrence prediction model. Among these three gene signatures, there was only one common gene, *BGN*, which was selected by both Oncotype and us. The remaining genes from these three biomarker panels were all different.

### Chemotherapy efficacy prediction

In total, 192 stage III CRC patients treated with 5FU were used for the efficacy study, of whom 119 did not experience recurrence during the follow-up period. Another set of 166 stage III patients treated with FOLFOX were used for the efficacy study, of whom 102 did not experience recurrence during the follow-up period. The efficacy of each drug was defined by the patients’ recurrence status. Responders were patients who had no recurrence for at least 48 months after the surgery, while non-responders were patients who experienced recurrence during the follow-up. Supplementary Table 2 lists the demographics of the patients in the chemotherapy study.

**Figure 3:**
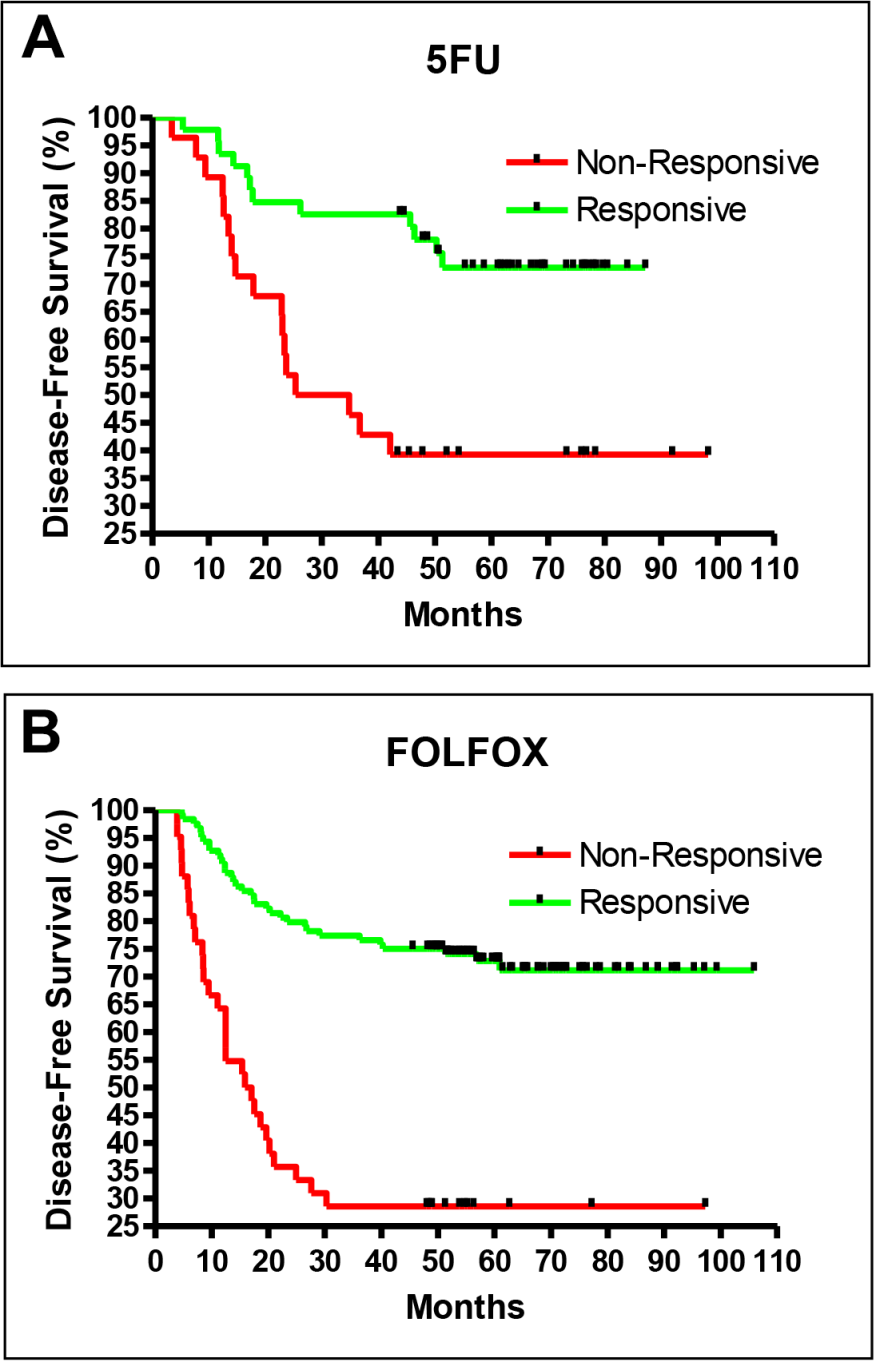
Drug efficacy prediction results for 5FU (**A**) and FOLFOX (**B**).

The training for these two drugs followed the two-stage procedure described above for the recurrence study. In the first stage, about one-third of the patients were reserved as the blind test set, and the remaining two-thirds were used as the training set. The training set was used to generate a prediction model, and the test set was used to validate the prediction model. The final performances of the prediction models were then determined with the use of all samples from both data sets and the leave-five-out iteration method during the second stage. The 5FU (Figure 3A) and FOLFOX (Figure 3B) prediction models both performed well, with excellent performance indicators (Table 2).

The sensitivity of the drug efficacy models was defined as the ability to detect patients who would benefit from adjuvant treatment (i.e., no recurrence after adjuvant treatment), in contrast to the first part of the study, where sensitivity was defined as the ability to detect patients who would relapse, so they could be targeted for adjuvant treatment. When the default cutoff of zero was used, the FOLFOX model had a sensitivity of 0.88 and a specificity of 0.47, while the 5FU model had a sensitivity of 0.76 and a specificity of 0.59 (Table 2).

For the purpose of choosing between FOLFOX or 5FU for adjuvant treatment, it will be necessary to have high specificity for the 5FU prediction and high sensitivity for the FOLFOX prediction. The default cutoff yielded a high sensitivity of 0.88 for FOLFOX prediction, whereas a cutoff score of -1.1 achieved a high specificity of 0.86 for 5FU prediction. With these cutoffs, only high-confidence 5FU responders would be treated with 5FU, while lower-confidence responders and non-responders to 5FU would be treated with FOLFOX.

## DISCUSSION

### Stage I and II CRC patients were chosen for the prognostic study

In published studies, it has been common for prediction models to perform differently for different stages of disease. The recurrence rate in stage I CRC patients is the lowest, and is the most difficult to predict accurately. In creating this prognostic prediction model, our objective was to achieve good performance for both stage I and II patients. Thus, samples from stage I and II patients were chosen for training and for determining the gene expression separation boundaries between high-risk and low-risk patients. Our results demonstrated that the model using targeted stage samples performed better than using non-targeted stage samples.

Some studies used samples from non-targeted stage I and IV patients (who exhibit a clean separation) to distinguish between targeted high-risk and low-risk stage II and III patients [20, 23, 27]. By this design, the true boundary between recurrent and non-recurrent patients could not be determined in these studies, since targeted stage II and III patients were not included in the training. This could explain the contradictory conclusions of different studies in which this approach was used; for instance, one model predicted recurrence for stage III patients but not for stage II patients [20], while another model predicted recurrence for stage II patients but not for stage III patients [27]. In a third study, recurrence could be predicted for both stage II and III patients; however, the results were obtained from two different platforms, so further validation of the models was needed [23].

### Binary training

In many gene studies, three classifications have been used for patients: high-risk, intermediate-risk, and low-risk. Since an intermediate-risk classification does not allow a clinician to make a clear treatment decision, a binary high-risk and low-risk classification separation was chosen for this study. The binary training process forces the prediction model to learn the gene expression separation boundaries between two classifications of samples. A binary-decision-based prediction model still allows low-confidence high-risk and low-risk patients to be reclassified as intermediate-risk after the training prediction model is made. Such reclassification would yield a much smaller intermediate-risk population than training without the constraint of forced binary classification.

### Larger numbers of genes are needed for heterogeneous CRC

Many prognostic recurrence studies have included a small number of biomarkers, such as gene [19, 25, 26], microRNA [18], or protein expression markers [21], and have had limited success. In fact, when published gene or protein markers were retested in a separate study, these markers failed to predict recurrence [21]. Due to the limited performance of prediction models, some investigators have added clinicopathologic variables to gene-based prediction models to enhance the overall system performance [48, 49]. However, the addition of clinicopathologic variables seems contrary to the basic notion of using genomic information for prediction. Conceptually, for a gene-based prognostic analysis to be robust, the relevant gene information related to clinicopathologic variables should be extracted to ensure that the prediction does not require information from clinical variables to be added externally.

The heterogeneity of cancer could be one factor that has limited the performance of studies in which smaller numbers of biomarkers were used. Marisa et al. [28] found a relationship between heterogeneity and recurrence when unsupervised hierarchical clustering of gene expression data was used to identify six molecular subtypes. Different subtypes were independently associated with different relapse-free survival times after adjustment for age, sex, and stage. Shibayama et al. reviewed all published prognostic models and found that there was little overlap among the gene lists. The authors cited the tumor heterogeneity of CRC as one of the reasons for this lack of overlap [30]. To overcome this heterogeneity issue, we chose to use microarrays so that a larger number of genes could be included in the models. In addition, the large number of genes detected by the microarray (most of which were not useful for the prediction) provided a stable baseline for calibrating the relevant genes used for prediction.

### Different gene lists among studies

The genes selected for our recurrence prediction model were very different from those selected by Oncotype and ColoPrint. When we tested our samples for each of the 18 ColoPrint genes, we were not able to distinguish between recurrent and non-recurrent samples, most likely because of the different platforms and sample types used (PCR and fresh-frozen samples for ColoPrint vs. microarray and FFPE in this study). On the other hand, Oncotype used PCR, as ColoPrint did, but used FFPE samples for training, as we did in this study. In addition to *BGN*, Oncotype genes *MK167* and *MYBL2* could also be used to distinguish between recurrent and non-recurrent samples in our dataset; however, their prediction performances were inferior to those of the other genes selected in our final model.

This issue of divergent gene lists generated from different platforms was previously examined by the FDA's Microarray Quality Control workshop. The disparity was found to be due to the different probes and labels used by each platform [50]. Tan et al. demonstrated that even when the same RNA samples were tested on three different commercial platforms, the resultant differentially expressed gene panels were very different [51]. The number of differentially expressed genes identified ranged from 34 to 113, and only four genes were commonly selected across all three platforms. Therefore, it is not surprising that the biomarker panels generated by ColoPrint, Oncotype, and our study were so disparate, as they were generated with different platforms and types of samples.

### Results of recurrence prediction

The present method of genomic study allowed us to generate a much-improved recurrence prediction model for all three types of CRC: right-sided colon cancer, left-sided colon cancer and rectal cancer. More importantly, the model was effective not only for stage II patients, but also for the more statistically challenging stage I patients. The consistency of performance for stage I and II patients over the three types of CRC indicated the robustness of this prediction model, and was an important improvement over other published results and the commercial services of Oncotype and ColoPrint.

The performance of our model was best represented by the high area under the curve of 0.77 for the Receiver Operating Characteristic curve. At the default cutoff value of 0, the sensitivity was 0.80, and the HR of recurrence in high-risk vs. low-risk CRC patients was 4.74. An important point is that the current results were generated by a forced binary decision approach. If the marginal patients between high-risk and low-risk patients were reclassified as intermediate-risk patients, the HR values would be higher. This HR value allows better identification of high-risk patients than those of the existing models. The five-year DFS difference between high-risk and low-risk patients was 46% (Figure 2A), about three times higher the DFS differences of Oncotype and ColoPrint.

### Results of chemotherapy efficacy prediction

The second part of this study addressed the problem of selecting the most effective adjuvant chemotherapeutic regimen. Currently, the efficacy of some chemotherapy drugs can be predicted by one or two markers; for instance, the efficacy of Cetuximab is predicted by KRAS/BRAF expression [52]. This approach is possible when the pathway information of a drug is known; however, this is not true for many drugs, especially cytotoxic therapeutics. At present, combination drug treatments including cytotoxic compounds are commonly used, and their efficacies depend on many factors and cellular pathways [47]. Even when the mechanism is understood, the prediction of efficacy is limited by knowledge of the specific pathway. For example, KRAS and BRAF are negative predictors (i.e., of who will not benefit from Cetuximab), but do not provide positive predictions of who will benefit from Cetuximab as part of a combination therapy. On the other hand, a whole-genome-based analysis can extract information from many unknown pathways in the training process. Pathway analysis is critical for new scientific discoveries, but is not as efficient as whole-genome analysis in capturing discriminate factors for phenotype classification.

In addition, chemotherapy efficacy prediction is more than just the ability to predict the efficacy of an individual drug. A critical component of treatment determinations is the ability to compare several drug efficacy predictions before selecting the optimal plan. In general, different prediction models are trained independently, and each model retains the characteristics of the training process. Since the conditions of each training are different, it is very difficult if not impossible to compare results from different models. We avoided this issue by constraining the training process to produce a pair of consistent 5FU and FOLFOX prediction models.

The efficacy of our chemotherapeutic prediction model is the best demonstration of the capability of full genomic analysis, especially for the complex combination drug regimen of FOLFOX. With the success of our chemotherapy efficacy prediction model, there is now a path toward developing a complete set of prediction models for early-stage CRC patients. Our prognostic prediction model can be used to select high-risk patients for adjuvant treatment, and the two chemotherapy prediction models can be used to select the proper adjuvant treatment and thus form a truly personalized treatment plan.

It should be noted that the chemotherapeutic efficacy model was developed with stage III CRC patients, since few stage I/II patients receive chemotherapy. If the drug prediction models generated from stage III patients are verified to be effective for stage I and II patients in prospective studies, we will have a set of predictions that can be used to form complete personalized treatment plans for early-stage CRC patients. For patients with more advanced stages of CRC, this approach can be used to evaluate additional chemotherapies in the future.

## MATERIALS AND METHODS

### Patients

This study, conducted at Taipei Veterans General Hospital, conformed to the guidelines of the ethics committee, and was approved by the Internal Review Board (VGHIRB Number: 2013-06-005AC). In total, 593 patients with pathological stage I – III CRC who had undergone R0 curative resection between March 2003 and November 2010 at Taipei Veterans General Hospital were enrolled. Clinical information was prospectively obtained and recorded in a computerized database, including patient demographics (age, gender, and comorbidities), tumor characteristics (location, TNM stage, differentiation, and prognostic features), and follow-up data. After surgery, patients were examined at an outpatient department every three months for the first two years, every six months for the third and fourth years, and annually thereafter. Follow-up examinations included serum CEA and CA19-9 level measurements, chest radiography, and abdominal ultrasonography. Abdominal/pelvic with- or without-chest computed tomography was performed annually and whenever recurrence was suspected. Colonoscopy was performed one year after surgery and every two to three years thereafter. If cecal intubation was not achieved preoperatively, a colonoscopy was performed three to six months after operation.

In total, 235 patients with pathological early-stage (I and II) CRC who had undergone complete resection were included in the recurrence study. None of them received neoadjuvant treatment. Two hundred and ten of them did not receive adjuvant treatment after surgery, while 25 received adjuvant treatment but experienced recurrence during follow-up. Stage III CRC patients who had received adjuvant treatment were used for the chemotherapy efficacy studies; 192 patients received 5FU and 166 patients received FOLFOX.

Most early-stage CRC patients will not experience recurrence; thus, a prediction model developed with the data from all available patients will perform well for non-recurrent patients but poorly for recurrent patients, due to the sampling bias introduced by the uneven numbers of non-recurrent and recurrent samples. In the current study, we selected comparable numbers of recurrent and non-recurrent patients to ensure that the prediction model developed would perform similarly for both groups.

Written informed consents for tissue collection were obtained from all patients. FFPE tissue blocks were retrieved from the Biobank of Taipei Veterans General Hospital. For each patient, the most representative part of the tumor, usually the solid part next to the center of the tumor with no necrotic tissue, was prospectively collected, processed, and stored in the Biobank of Taipei Veterans General Hospital. Samples were examined by an expert pathologist (TY Chou), who determined the percentage of tumor cells; only samples with > 40% tumor cells were included in this study.

### Platform

We analyzed samples with the GeneChip^®^ Human ST 2.0 microarray (Affymetrix, Santa Clara, CA, USA), a whole-transcript array that includes probes to measure 30,654 mRNA and 11,086 long intergenic non-coding RNA (lincRNA) transcripts. The complete dataset (GSE81653) can be accessed at the NCBI Gene Expression Omnibus.

### Data generation

Total RNA was extracted from 10-um FFPE tissue sections by means of QIAsymphony RNA kits. All tissue samples had the minimum 40% tumor cell percentage. RNA 6000 Nano kits (Agilent, Santa Clara, CA, USA) were used to check the quality of the total RNA. The RNA quality was safeguarded with a cutoff value zero of delta Ct against 18S with qPCR. The Ovation Pico WTA System (Nugen, San Carlos, CA, USA) was used to amplify cDNA from total RNA. MinElute Reaction CleanUP Kits (Qiagen, Germantown, MD, USA) were used for purification, while the Encore Biotin Module (Nugen) was used for fragmentation and labeling. The fragmented, end-labeled cDNA samples were applied to the Affymetrix 2.0 ST arrays. The arrays were washed and stained with the GeneChip Fluidics Station 450.

### Data analysis

Gene expression data were extracted from the Affymetrix CEL data file and normalized with the vendor's robust multi-array average (RMA) software. The quality of labeling and hybridization was monitored with vendor-specified spikes. Their values and the values of additional quality controls provided by the vendor's Expression Console were within the specifications, ensuring the quality of the sample processing and gene expression data.

A supervised clustering method of KNN was used to analyze the gene expression data and generate a prediction model. Unknown samples were categorized as high-risk or low-risk depending on the classification of the KNN. The distance used to measure the closeness between samples was the correlation of their mRNA levels. A *t-test* was used to select the top 500 genes that best separated high-risk and low-risk samples. The final genes chosen for prediction (Supplementary Table 3) and the optimal values of K (1-3) of KNN for the prediction were determined by 20-fold training and testing iterations among samples.

For each sample, the prediction model yielded a prediction score based on a particular cutoff value for a binary high-risk vs. low-risk decision. The positive or negative sign of the score indicated a high-risk or low-risk prediction, respectively. A larger absolute value (e.g., score > 0.5 or score < –0.5) of the score indicated a greater confidence in the prediction, while a smaller absolute value (e.g., –0.5 < score < 0.5) of the score indicated a lower-confidence classification. The default cutoff between a positive and a negative decision was zero, but other cutoff values could be used to trade sensitivity for specificity.

### Blind validation testing

A two-stage method was used to generate each of the three prediction models. In the first stage, one-third of the samples were set aside as the blind test set with no clinical information available. The remaining samples in the training set had unblinded clinical information to allow for the generation of the prediction models. After the training, the performance of the prediction model was determined with the blind test set. After the model yielded the prediction results for the previously reserved blind samples, only then were the clinical statuses of those samples revealed so that the prediction accuracy of the model could be calculated.

After the validation of the first-stage training process, no programming modifications were made to the model during the second stage of model generation. This ensured that no new bias was introduced. During the second stage, the training set and blind test sets were studied together, and a leave-five-out iteration method was used to calculate the final performance.

### Biomarkers used in commercial services

Oncotype and ColoPrint provide commercial services to predict the recurrence of stage II CRC patients. Oncotype uses a 12-gene assay, including seven cancer-related genes (*BGN, MKI67, MYBL2, GADD45B, FAP, INHBA*, and *C-MYC*) and five reference genes (*ATP5E, GPX1, PGK1, UBB*, and *VDAC2*). ColoPrint uses an 18-gene assay, including *MCTP1, LAMA3, CTSC, PYROXD1, EDEM1, IL2RB, ZNF697, SLC6A11, IL2RA, CYFIP2, PIM3, LIF, PLIN3, HSD3B1, ZBED4, PPARA, THNSL2*, and *CA438802*.

### Performance analysis

The performance of the prediction model was characterized with the following measurements: the HR of recurrence in the predicted high-risk group vs. the low-risk group; the sensitivity; the specificity; and the area under the curve of the Receiver Operating Characteristic curve. The system provided binary prediction results for all samples, i.e., there was not an intermediate-risk classification for the predictions. The HRs and curves were generated by the Kaplan-Meier method, and the comparison between the curves was performed with the log-rank test. All calculations were conducted in GraphPad's PRISM software.

### Statistical analysis

The group distributions for each clinicopathological trait were compared through a two-tailed Fisher's exact procedure and the chi-square test. Numerical values were compared through Student's *t*-test. Data are expressed as the mean ± standard deviation. Multivariate analysis was performed with the Cox proportional hazard model. Statistical significance was defined as *P* < 0.05. Statistical analyses were performed with the SPSS package (version 16.0 for Windows, SPSS, Chicago, IL, USA).

## SUPPLEMENTARY MATERIALS FIGURES AND TABLES






