# Supplementary material for: Building personalized treatment plans for early-stage colorectal cancer patients
Source: Oncotarget. 2017 Jan 13;8(8):13805–17. doi: 10.18632/oncotarget.14638 (PMC5355140; doi:10.18632/oncotarget.14638)
Supplement: Supplementary file 1 [file oncotarget-08-13805-s001.pdf]

Building personalized treatment plans for early-stage colorectal cancer patients

Supplementary Materials

Supplementary Table 1: Univariate and multivariate analysis of potential prognostic factors for 5-year disease-free survival for all recurrence study patients

Variable	Univariate			Multivariate	
	No. of patients	5-year DFS (%)	*p*	HR	*p*
Gender			0.558		
Male	145	51.1			
Female	90	58.2			
Location			0.123		
Right colon	68	59.8			
Left colon	106	56.9			
Rectum	61	41.7			
Preoperative CEA level			< 0.001	1.70	0.005
< 5 ng/mL	160	63.2			
> 5 ng/mL	64	35.8			
Stage			0.572		
I	39	58.2			
II	196	53.5			
Emergent operation			0.036		0.794
No	225	54.8			
Yes	10	30.0			
Mucinous component (> 50%)			0.641		
No	228	66.7			
Yes	7	53.4			
Lymphovascular invasion			0.012		0.489
No	122	55.1			
Yes	13	30.8			
Perineural invasion			0.576		
No	229	53.6			
Yes	6	55.6			
Grade of differentiation			0.438		
Well/moderately	231	53.3			
Poorly/undifferentiated	4	50.0			
Lymph nodes harvested			0.549		
≥ 12	176	55.8			
< 12	59	47.8			
Adjuvant chemotherapy			< 0.001	2.50	0.001
Yes	25	19.6			
No	210	58.3			

Abbreviations: CEA, carcinoembryonic antigen; DFS, disease-free survival.

Supplementary Table 2: Demographics of patients with chemotherapy study

Variable	Total		5-FU		FOLFOX	
	***n* = 358**	**%**	***n* = 192**	**%**	***n* = 166**	**%**
Age (Mean ± SD)	62.8 ± 12.0		65.0 ± 11.8		60.2 ± 11.7	
Follow-up (Months ± SD)	59.0 ± 26.9		67.0 ± 28.0		50.7 ± 22.5	
Gender						
Male	215	60.1	116	60.4	99	60.0
Female	143	39.9	76	39.6	67	40.0
Location						
Right colon	103	28.8	54	28.0	49	29.5
Left colon	140	39.1	69	36.0	71	42.8
Rectum	115	32.1	69	36.0	46	27.7
Preoperative CEA level						
< 5 ng/mL	235	65.6	127	66.2	108	65.1
> 5 ng/mL	104	29.1	54	28.1	50	30.1
NA	19	5.3	11	5.7	8	4.8
Emergent operation						
No	338	94.4	181	94.3	157	94.6
Yes	20	5.6	11	5.7	9	5.4
Mucinous component (> 50%)						
No	334	93.3	180	93.7	154	92.8
Yes	24	6.7	12	6.3	12	7.2
Lymphovascular invasion						
No	258	72.1	151	78.6	107	64.5
Yes	100	27.9	41	21.4	59	35.5
Perineural invasion						
No	336	93.9	183	95.3	153	92.2
Yes	22	6.1	9	4.7	13	7.8
Grade of differentiation						
Well/moderate	321	89.7	178	92.7	143	86.1
Poor/undifferentiated	37	10.3	14	7.3	23	13.9
Lymph nodes harvested						
≥ 12	310	86.6	158	82.3	152	91.6
< 12	48	13.4	34	17.7	14	8.4
Recurrence						
Yes	137	38.3	73	38.0	64	38.6
No	221	61.7	119	62.0	102	61.4

Abbreviations: CEA, carcinoembryonic antigen.

Supplementary Table 3: Gene list. See Supplementary_Table_3

Supplementary Table 4: Prediction score. See Supplementary_Table_4
