# Supplementary material for: Building personalized treatment plans for early-stage colorectal cancer patients
Source: Oncotarget. 2017 Jan 13;8(8):13805–17. doi: 10.18632/oncotarget.14638 (PMC5355140; doi:10.18632/oncotarget.14638)
Supplement: Supplementary file 2 [file oncotarget-08-13805-s002.docx]

**Supplementary Table 3 Gene List**

Genes used in recurrence prediction

| probeset_id | seqname | start | stop | gene assignment |
| --- | --- | --- | --- | --- |
| 16760981 | chr12 | 9092957 | 9102551 | NM_002355.3//M6PR |
| 16852331 | chr18 | 48405419 | 48476165 | NM_002396.4//ME2 |
| 16673410 | chr1 | 167690429 | 167761156 | ENST00000487858//MPZL1 |
| 16693280 | chr1 | 152004982 | 152020383 | NM_005620.1//S100A11 |
| 17049676 | chr7 | 100770370 | 100782547 | NM_000602.3//SERPINE1 |
| 16858210 | chr19 | 10713121 | 10755235 | NM_020428.3//SLC44A2 |
| 16698049 | chr1 | 202696526 | 202778598 | NM_006618.3//KDM5B |
| 17126014 | gi337756904 | 1 | 68 | NR_039668.1 |
| 16831388 | chr17 | 15902694 | 15948336 | NM_017775.3//TTC19 |
| 16742838 | chr11 | 82789752 | 82790924 | XR_111199.1 |
| 17118154 | chr6 | 17759474 | 17760043 | XR_112825.2 |
| 16693898 | chr1 | 154934774 | 154946959 | NM_003029.4//SHC1 |
| 17029226 | chr6_cox_hap2 | 3204432 | 3217149 | NM_013974.1//DDAH2 |
| 17047459 | chr7 | 75573100 | 75573234 | NR_002955.1 |
| 16742286 | chr11 | 75282944 | 75283832 | ENST00000525418//RP11-939C17.2.1 |
| 17120868 | TCONS_l2_00006703 | 1 | 673 | TCONS_l2_00006703 |
| 16762280 | chr12 | 22613453 | 22616848 | ENST00000543604//RP11-359J14.2.1 |
| 17015919 | chr6 | 17759414 | 17987854 | NM_001105568.2//KIF13A |
| 17042023 | chr6_ssto_hap7 | 3029175 | 3038358 | NM_001288.4//CLIC1 |
| 17036745 | chr6_mcf_hap5 | 3078041 | 3087224 | NM_001288.4//CLIC1 |
| 17039540 | chr6_qbl_hap6 | 2991988 | 3001178 | NM_001288.4//CLIC1 |
| 16929416 | chr22 | 32870663 | 32894818 | NM_012179.3//FBXO7 |
| 16693789 | chr1 | 154521051 | 154531504 | NM_017582.6//UBE2Q1 |
| 17108003 | chrX | 152760347 | 152775012 | NM_001711.4//BGN |
| 17011302 | chr6 | 106808784 | 107018335 | NM_001624.2//AIM1 |
| 16686293 | chr1 | 45271580 | 45272957 | NM_001013632.2//TCTEX1D4 |
| 17124060 | TCONS_l2_00023407 | 1 | 1073 | TCONS_l2_00023407 |
| 16950912 | chr3 | 12938719 | 13114617 | NM_014869.4//IQSEC1 |
| 17032027 | chr6_dbb_hap3 | 2983927 | 2993114 | NM_001288.4//CLIC1 |
| 16866562 | chr19 | 1037312 | 1039056 | XR_133162.1 |
| 17025844 | chr6 | 169615875 | 169654139 | NM_003247.2//THBS2 |
| 16782187 | chr14 | 23305766 | 23318236 | NM_004995.2//MMP14 |
| 16710126 | chr10 | 124221041 | 124274424 | NM_002775.4//HTRA1 |
| 16855491 | chr18 | 55267891 | 55289177 | NM_004539.3//NARS |
| 17031373 | chr6_dbb_hap3 | 826740 | 831053 | NM_006398.3//UBD |
| 17122510 | TCONS_l2_00014805 | 1 | 501 | TCONS_l2_00014805 |
| 16761631 | chr12 | 12626216 | 12715448 | NM_030640.2//DUSP16 |
| 16737759 | chr11 | 46698630 | 46722165 | NM_004308.2//ARHGAP1 |
| 17017370 | chr6 | 31694815 | 31707540 | ENST00000375787//DDAH2 |
| 17004208 | chr6 | 1610679 | 1614132 | NM_001453.2//FOXC1 |
| 17122506 | TCONS_l2_00014803 | 1 | 492 | TCONS_l2_00014803 |
| 16986913 | chr5 | 82767284 | 82878122 | NM_004385.4//VCAN |
| 17017924 | chr6 | 32717689 | 32717756 | NR_039668.1 |
| 17042431 | chr6_ssto_hap7 | 4149356 | 4149423 | NR_039668.1 |
| 17027089 | chr6_apd_hap1 | 4005968 | 4006035 | NR_039668.1 |
| 17029733 | chr6_cox_hap2 | 4163592 | 4163659 | NR_039668.1 |
| 17034497 | chr6_mann_hap4 | 3041248 | 3050430 | NM_001288.4//CLIC1 |
| 16937024 | chr3 | 5020801 | 5027008 | NM_003670.2//BHLHE40 |
| 17103185 | chrX | 47441690 | 47446190 | NM_003254.2//TIMP1 |
| 16769039 | chr12 | 100550169 | 100567121 | NR_036632.1 |
| 16691383 | chr1 | 116956388 | 116956491 | ENST00000391083//U6 |
| 16888610 | chr2 | 189839046 | 189877472 | NM_000090.3//COL3A1 |
| 17001927 | chr5 | 151040657 | 151066726 | XR_133412.1 |
| 16673056 | chr1 | 162531296 | 162569633 | NM_003115.4//UAP1 |
| 16932606 | chr22 | 20829490 | 20829598 | ENST00000383946//Y_RNA |
| 16799814 | chr15 | 41700606 | 41775761 | ENST00000558298//RTF1 |
| 17006554 | chr6 | 31082527 | 31107869 | ENST00000548049//PSORS1C1 |
| 16968680 | chr4 | 88896802 | 88904563 | NM_001251829.1//SPP1 |
| 16669783 | chr1 | 145291855 | 145291954 | ENST00000479995//NOTCH2NL |
| 16846587 | chr17 | 48260650 | 48279000 | NM_000088.3//COL1A1 |
| 16847260 | chr17 | 57774667 | 57784987 | NM_016077.3//PTRH2 |
| 16746992 | chr12 | 4382902 | 4414522 | NM_001759.3//CCND2 |
| 16827041 | chr16 | 64977656 | 65156101 | NM_001797.2//CDH11 |
| 17102861 | chrX | 44732423 | 44971847 | NM_021140.2//KDM6A |
| 17022929 | chr6 | 116440085 | 116479910 | NM_000493.3//COL10A1 |
| 16746857 | chr12 | 2939591 | 2969202 | NR_038933.1 |
| 16883498 | chr2 | 101436613 | 101613291 | ENST00000427413//NPAS2 |
| 16855697 | chr18 | 61056423 | 61089752 | NM_004869.3//VPS4B |
| 16852354 | chr18 | 48556583 | 48611415 | NM_005359.5//SMAD4 |
| 16846398 | chr17 | 47366568 | 47439835 | NM_014897.2//ZNF652 |
| 16789178 | chr14 | 103799881 | 103811362 | NM_001969.3//EIF5 |
| 16839973 | chr17 | 4062960 | 4063932 | TCONS_00026020//linc-ZZEF1-1 |
| 17007837 | chr6 | 35436178 | 35438562 | ENST00000464112//RPL10A |
| 17122508 | TCONS_l2_00014804 | 1 | 396 | TCONS_l2_00014804 |
| 16693546 | chr1 | 153777201 | 153895451 | NM_020699.2//GATAD2B |
| 17048473 | chr7 | 94023873 | 94060544 | NM_000089.3//COL1A2 |
| 16676643 | chr1 | 206858289 | 206907630 | NM_004759.4//MAPKAPK2 |
| 16752715 | chr12 | 57522276 | 57607142 | NM_002332.2//LRP1 |
| 16937563 | chr3 | 10157276 | 10168874 | NM_018462.4//BRK1 |
| 16856581 | chr19 | 1438363 | 1440492 | NM_001018.3//RPS15 |
| 16991246 | chr5 | 151041996 | 151043043 | XR_133413.1 |
| 17065047 | chr7_gl000195_random | 42938 | 86719 | NM_001242480.1//LOC389831 |
| 16693959 | chr1 | 155141884 | 155145951 | NM_173852.3//KRTCAP2 |
| 16743890 | chr11 | 104895996 | 104972158 | NM_033294.2//CASP1 |
| 16837284 | chr17 | 66097696 | 66132070 | TCONS_l2_00010933 |
| 17121834 | TCONS_l2_00011427 | 1 | 12147 | TCONS_l2_00011427 |
| 16715389 | chr10 | 74885838 | 74885965 | ENST00000408237//SNORA11 |
| 16793748 | chr14 | 64118015 | 64118217 | ENST00000363823//U3 |
| 16879923 | chr2 | 48010221 | 48034092 | NM_000179.2//MSH6 |
| 16778241 | chr13 | 38136282 | 38183563 | NM_006475.2//POSTN |
| 16909828 | chr2 | 238232646 | 238323018 | NM_004369.3//COL6A3 |
| 17012804 | chr6 | 135502453 | 135540311 | ENST00000531845//MYB |
| 16949839 | chr3 | 195343316 | 195467994 | NM_152673.2//MUC20 |
| 16980762 | chr4 | 154701742 | 154710272 | NM_003013.2//SFRP2 |
| 16885978 | chr2 | 136289025 | 136482840 | NM_015361.2//R3HDM1 |
| 17125620 | TCONS_l2_00030521 | 1 | 455 | TCONS_l2_00030521 |
| 17057460 | chr7 | 45143948 | 45144081 | NR_002919.1 |
| 16989496 | chr5 | 135364584 | 135399507 | NM_000358.2//TGFBI |
| 16801143 | chr15 | 52121825 | 52239492 | NM_014547.4//TMOD3 |
| 16662584 | chr1 | 36771988 | 36794818 | NM_024676.4//SH3D21 |
| 16964098 | chr4 | 1976363 | 1976487 | NR_003004.1 |
| 16774112 | chr13 | 38923942 | 38937143 | NM_016617.2 |
| 17118430 | chr9 | 137726713 | 137736686 | XR_109855.2 |
| 16672583 | chr1 | 160175125 | 160185464 | NM_003768.3//PEA15 |
| 16686060 | chr1 | 43391046 | 43424847 | NM_006516.2//SLC2A1 |
| 17105706 | chrX | 102611373 | 102613397 | NM_001006612.1//WBP5 |
| 17009093 | chr6 | 43737921 | 43754224 | NM_001025366.2//VEGFA |
| 17118732 | tc5006997 | 1 | 80 | NR_002579.1 |
| 16742384 | chr11 | 76368568 | 76381791 | NM_005512.2//LRRC32 |
| 17018385 | chr6 | 34254567 | 34393902 | NM_006703.3//NUDT3 |
| 16960114 | chr3 | 145787227 | 145881440 | NM_182943.2//PLOD2 |
| 16829777 | chr17 | 4063018 | 4063996 | TCONS_00025805//linc-SPNS3-1 |
| 16669169 | chr1 | 117297007 | 117311851 | NM_001767.3//CD2 |
| 16853418 | chr18 | 670324 | 712676 | NM_001126123.3//ENOSF1 |
| 17121836 | TCONS_l2_00011429 | 1 | 4597 | TCONS_l2_00011429 |
| 16774623 | chr13 | 47127296 | 47327176 | NM_015116.2//LRCH1 |
| 17089371 | chr9 | 130374486 | 130457460 | NM_001032221.3//STXBP1 |
| 16809929 | chr15 | 59171244 | 59225852 | NM_001013843.1//SLTM |
| 16778392 | chr13 | 41129801 | 41240734 | NM_002015.3//FOXO1 |
| 16771242 | chr12 | 120648242 | 120703574 | NM_001080855.2//PXN |

Genes used in 5FU efficacy prediction

| probeset_id | seqname | start | stop | gene assignment |
| --- | --- | --- | --- | --- |
| 17032402 | chr6_dbb_hap3 | 3897030 | 3897935 | ENST00000550466//CR753846.2 |
| 16869324 | chr19 | 12907634 | 12912694 | NM_005809.4//PRDX2 |
| 17125592 | TCONS_l2_00030421 | 1 | 486 | TCONS_l2_00030421 |
| 16758879 | chr12 | 125478194 | 125515684 | NM_080626.5//BRI3BP |
| 16965773 | chr4 | 28821204 | 28821290 | NR_036237.1 |
| 16664003 | chr1 | 45242162 | 45242265 | NR_000024.2 |
| 16702547 | chr10 | 13141449 | 13180291 | ENST00000263036//OPTN |
| 17118724 | tc5006985 | 1 | 85 | NR_003939.1 |
| 16679981 | chr1 | 808847 | 808957 | ENST00000408219 |
| 16906654 | chr2 | 196535096 | 196535198 | ENST00000364943//U6 |
| 16679887 | chr1 | 134773 | 140566 | XM_003403549.1//LOC100653346 |
| 16850426 | chr18 | 48162 | 48272 | ENST00000408514//AP001005.1 |
| 16980762 | chr4 | 154701742 | 154710272 | NM_003013.2//SFRP2 |
| 16894894 | chr2 | 20232411 | 20251789 | NM_014713.4//LAPTM4A |
| 17077135 | chr8 | 53535016 | 53658403 | NM_014781.4//RB1CC1 |
| 16854145 | chr18 | 18529701 | 18691812 | NM_005406.2//ROCK1 |
| 16837326 | chr17 | 66508110 | 66529147 | NM_212471.1//PRKAR1A |
| 17095887 | chr9 | 95218487 | 95244844 | NM_017680.4//ASPN |
| 16957884 | chr3 | 120111140 | 120170100 | NM_007085.4//FSTL1 |
| 16671545 | chr1 | 154975106 | 154991001 | NM_001252406.1//ZBTB7B |
| 16859696 | chr19 | 18082834 | 18082926 | ENST00000516782//5S_rRNA |
| 17095041 | chr9 | 77796436 | 77796537 | ENST00000363171//Y_RNA |
| 16797375 | chr14 | 106185955 | 106188274 | ENST00000558023//AL901608.1 |
| 17114567 | chrX | 135961358 | 135961430 | NR_002735.1 |
| 17075829 | chr8 | 28203102 | 28260218 | NM_018660.2//ZNF395 |
| 16897834 | chr2 | 56093097 | 56151298 | NM_001039348.2//EFEMP1 |
| 17020317 | chr6 | 56322785 | 56820047 | NM_015548.4//DST |
| 17125972 | gi337756556 | 1 | 80 | NR_039784.1 |
| 16828450 | chr16 | 75327608 | 75467387 | NM_006324.2//CFDP1 |
| 17076694 | chr8 | 41786997 | 41909508 | NM_001099412.1//KAT6A |
| 16732106 | chr11 | 118914899 | 118916205 | ENST00000531886//RP11-110I1.6.1 |
| 16684192 | chr1 | 28905255 | 28905334 | NR_003077.1 |
| 16868138 | chr19 | 8376184 | 8386280 | NM_005001.3//NDUFA7 |
| 17054527 | chr7 | 1881252 | 1881812 | ENST00000428925//AC110781.5.1 |
| 16977833 | chr4 | 88394487 | 88452213 | NM_001128310.1//SPARCL1 |
| 16686271 | chr1 | 45187458 | 45187574 | NR_002753.5 |
| 16971048 | chr4 | 144434616 | 144478642 | NM_003601.3//SMARCA5 |
| 16963646 | chr3 | 197846768 | 197846878 | ENST00000408525//AC073135.1 |
| 16761820 | chr12 | 15034115 | 15038860 | NM_000900.3//MGP |
| 16967187 | chr4 | 60698893 | 60698994 | ENST00000364806//Y_RNA |
| 16896910 | chr2 | 42989639 | 42991401 | NM_148962.4//OXER1 |
| 16808974 | chr15 | 49398268 | 49447858 | ENST00000560240//COPS2 |
| 16701689 | chr10 | 93769 | 93877 | ENST00000408830//AL713922.1 |
| 16958702 | chr3 | 126283408 | 126283521 | ENST00000365098//RN5S138//RNA, |
| 16866951 | chr19 | 2427636 | 2456994 | NM_032737.2//LMNB2 |
| 16674805 | chr1 | 182992595 | 183114727 | NM_002293.3//LAMC1 |
| 17119610 | tc5271504 | 1 | 301 | ENST00000548509//AL773544.4 |
| 16996345 | chr5 | 55556160 | 55556273 | ENST00000390963//RN5S185 |
| 16730104 | chr11 | 93454679 | 93455032 | NR_002569.2 |
| 16866709 | chr19 | 1576678 | 1592710 | NM_003926.5//MBD3 |
| 16726188 | chr11 | 63753325 | 63769283 | NM_017670.2//OTUB1 |
| 16838879 | chr17 | 79670400 | 79674556 | NM_002949.3//MRPL12 |
| 16683644 | chr1 | 25548767 | 25559013 | NM_207170.3//SYF2 |
| 16700074 | chr1 | 227177566 | 227506175 | NM_003607.3//CDC42BPA |
| 17000563 | chr5 | 138700366 | 138705406 | ENST00000503553//CTB-43P18.1.1 |
| 16914755 | chr20 | 48411348 | 48411451 | ENST00000516731//U6 |
| 16705356 | chr10 | 70090931 | 70102953 | ENST00000461310//HNRNPH3 |
| 17039221 | chr6_qbl_hap6 | 2399704 | 2401082 | ENST00000548509//AL773544.4 |
| 16829369 | chr16 | 89803959 | 89883065 | NM_000135.2//FANCA |
| 16904039 | chr2 | 160175490 | 160473203 | NM_013450.2//BAZ2B |
| 16976211 | chr4 | 57896939 | 57976551 | NM_001553.2//IGFBP7 |
| 16993197 | chr5 | 177388397 | 177388943 | TCONS_00010852//linc-FAM153C-2 |
| 17118180 | chr6 | 132269313 | 132269801 | XR_110171.1 |
| 16803185 | chr15 | 75628232 | 75634268 | NM_017828.3//COMMD4 |
| 16964098 | chr4 | 1976363 | 1976487 | NR_003004.1 |
| 16918419 | chr20 | 32244893 | 32262269 | NM_031232.3//NECAB3 |
| 17097464 | chr9 | 116135698 | 116139279 | NM_031219.2//HDHD3 |
| 16968719 | chr4 | 89122855 | 89122962 | ENST00000364837//U6 |
| 16897637 | chr2 | 55199325 | 55339757 | NM_020532.4//RTN4 |
| 16790233 | chr14 | 21677295 | 21737653 | NM_031314.2//HNRNPC |
| 16889636 | chr2 | 203241050 | 203432474 | NM_001204.6//BMPR2 |
| 17112269 | chrX | 76760356 | 77041719 | NM_000489.3//ATRX |
| 17100614 | chr9 | 141070834 | 141070938 | ENST00000408357//AL591424.1 |
| 16668712 | chr1 | 112084840 | 112259313 | ENST00000356415//RAP1A |
| 16730965 | chr11 | 108876244 | 108876350 | ENST00000364373//U6 |
| 17119534 | tc5266008 | 1 | 63 | NR_002745.1 |
| 16673229 | chr1 | 164621133 | 164868533 | ENST00000558796//PBX1 |
| 17119496 | tc5263075 | 1 | 63 | NR_002745.1 |
| 17119596 | tc5270919 | 1 | 63 | NR_002745.1 |
| 16916012 | chr20 | 62289163 | 62330051 | NM_016434.3//RTEL1 |
| 16691270 | chr1 | 115259534 | 115301297 | NM_007158.5//CSDE1 |
| 17068093 | chr8 | 38585704 | 38710546 | NM_001146216.2//TACC1 |
| 17079210 | chr8 | 95261481 | 95274578 | NM_005261.3//GEM |
| 16817647 | chr16 | 29802041 | 29816706 | ENST00000400751//KIF22 |
| 16962185 | chr3 | 183960089 | 183967336 | NM_005787.5//ALG3 |
| 16940108 | chr3 | 45633747 | 45633819 | ENST00000516118//AC099539.1 |
| 17017697 | chr6 | 32083041 | 32096030 | NM_004381.4//ATF6B |
| 16739543 | chr11 | 62539101 | 62559493 | ENST00000527073//TMEM223 |
| 16703036 | chr10 | 20105168 | 20569286 | NM_032812.7//PLXDC2//Homosapie |
| 16884280 | chr2 | 110744640 | 110753717 | TCONS_l2_00015441 |
| 16775189 | chr13 | 61187742 | 61187868 | ENST00000411144//RN5S31 |
| 16845899 | chr17 | 43471264 | 43507645 | NM_199282.2//ARHGAP27 |
| 16714084 | chr10 | 50193557 | 50193632 | NR_036182.1 |
| 17058249 | chr7 | 66452664 | 66460635 | NM_016038.2//SBDS |
| 17000808 | chr5 | 140050379 | 140053171 | NM_194249.2//DND1 |
| 16858092 | chr19 | 10216899 | 10226065 | ENST00000393793//PPAN |
| 16900441 | chr2 | 96808905 | 96811179 | NM_004418.3//DUSP2 |
| 17054474 | chr7 | 1778266 | 1781940 | XR_112968.1 |
| 17058601 | chr7 | 73183327 | 73184600 | NM_001306.3//CLDN3 |
| 16831383 | chr17 | 15848231 | 15879210 | NM_000676.2//ADORA2B |
| 16664599 | chr1 | 51701943 | 51739127 | NM_014372.4//RNF11 |

Genes used in FOLFOX efficacy prediction

| probeset_id | seqname | start | stop | gene assignment |
| --- | --- | --- | --- | --- |
| 16869324 | chr19 | 12907634 | 12912694 | NM_005809.4//PRDX2 |
| 16866951 | chr19 | 2427636 | 2456994 | NM_032737.2//LMNB2 |
| 17032402 | chr6_dbb_hap3 | 3897030 | 3897935 | ENST00000550466//CR753846.2 |
| 16758879 | chr12 | 1.25E+08 | 125515684 | NM_080626.5//BRI3BP |
| 16730965 | chr11 | 1.09E+08 | 108876350 | ENST00000364373//U6 |
| 17095041 | chr9 | 77796436 | 77796537 | ENST00000363171//Y_RNA |
| 16868138 | chr19 | 8376184 | 8386280 | NM_005001.3//NDUFA7 |
| 16837418 | chr17 | 70117161 | 70122561 | NM_000346.3//SOX9 |
| 17114567 | chrX | 1.36E+08 | 135961430 | NR_002735.1 |
| 16896910 | chr2 | 42989639 | 42991401 | NM_148962.4//OXER1 |
| 16759395 | chr12 | 1.32E+08 | 132428406 | NM_001002019.2//PUS1 |
| 16829369 | chr16 | 89803959 | 89883065 | NM_000135.2//FANCA |
| 16937503 | chr3 | 10028595 | 10046944 | XR_112457.1 |
| 17054474 | chr7 | 1778266 | 1781940 | XR_112968.1 |
| 16734281 | chr11 | 1605572 | 1606513 | NM_001005922.1//KRTAP5-1 |
| 17118650 | tc4982286 | 1 | 74 | NR_000015.2 |
| 16737841 | chr11 | 46783939 | 46784049 | NR_003056.1 |
| 16962185 | chr3 | 1.84E+08 | 183967336 | NM_005787.5//ALG3 |
| 17077135 | chr8 | 53535016 | 53658403 | NM_014781.4//RB1CC1 |
| 16967853 | chr4 | 75310851 | 75320726 | NM_001657.2//AREG |
| 17119488 | tc5262293 | 1 | 132 | NR_002960.1 |
| 16664003 | chr1 | 45242162 | 45242265 | NR_000024.2 |
| 17095887 | chr9 | 95218487 | 95244844 | NM_017680.4//ASPN |
| 16761820 | chr12 | 15034115 | 15038860 | NM_000900.3//MGP |
| 17090646 | chr9 | 1.36E+08 | 135570342 | NM_012204.2//GTF3C4 |
| 16966159 | chr4 | 39549456 | 39551432 | ENST00000412595//RP11-472B18.2 |
| 16970001 | chr4 | 1.16E+08 | 115599380 | NM_001128174.1//UGT8 |
| 16719515 | chr10 | 1.3E+08 | 129924649 | NM_002417.4//MKI67 |
| 16684192 | chr1 | 28905255 | 28905334 | NR_003077.1 |
| 16965773 | chr4 | 28821204 | 28821290 | NR_036237.1 |
| 16808149 | chr15 | 43825660 | 43982283 | NM_001190214.1//PPIP5K1 |
| 17068093 | chr8 | 38585704 | 38710546 | NM_001146216.2//TACC1 |
| 16838879 | chr17 | 79670400 | 79674556 | NM_002949.3//MRPL12 |
| 16732106 | chr11 | 1.19E+08 | 118916205 | ENST00000531886//RP11-110I1.6.1 |
| 16710015 | chr10 | 1.24E+08 | 123606215 | ENST00000365014//Y_RNA |
| 16977833 | chr4 | 88394487 | 88452213 | NM_001128310.1//SPARCL1 |
| 17119404 | tc5227702 | 1 | 85 | ENST00000516372//AC020703.1 |
| 16980768 | chr4 | 1.55E+08 | 154794603 | ENST00000516372//AC020703.1 |
| 16805218 | chr15 | 93425937 | 93441977 | NR_037600.1 |
| 16976211 | chr4 | 57896939 | 57976551 | NM_001553.2//IGFBP7 |
| 16937498 | chr3 | 9989088 | 9996471 | ENST00000431558//PRRT3-AS1 |
| 16686271 | chr1 | 45187458 | 45187574 | NR_002753.5 |
| 16817017 | chr16 | 23690143 | 23701688 | NM_005030.3//PLK1 |
| 16980762 | chr4 | 1.55E+08 | 154710272 | NM_003013.2//SFRP2 |
| 16784412 | chr14 | 56025790 | 56168244 | NM_001079521.1//KTN1 |
| 16837433 | chr17 | 71188771 | 71204646 | ENST00000438720//COG1 |
| 17058249 | chr7 | 66452664 | 66460635 | NM_016038.2//SBDS |
| 16913957 | chr20 | 42295709 | 42345136 | NM_002466.2//MYBL2 |
| 16679981 | chr1 | 808847 | 808957 | ENST00000408219 |
| 16743628 | chr11 | 1.02E+08 | 102101653 | ENST00000526310//RP11-864G5.3.1 |
| 17072766 | chr8 | 1.33E+08 | 133025889 | NM_015137.4//EFR3A |
| 16967863 | chr4 | 75480629 | 75490486 | ENST00000380846//AREGB |
| 16840018 | chr17 | 4442191 | 4458681 | NM_014520.3//MYBBP1A |
| 16863652 | chr19 | 48216601 | 48246391 | NM_014601.3//EHD2 |
| 17020317 | chr6 | 56322785 | 56820047 | NM_015548.4//DST |
| 16828108 | chr16 | 71792305 | 71792390 | NR_003059.1 |
| 16763032 | chr12 | 32943679 | 33049780 | NM_001005242.2//PKP2 |
| 16798154 | chr15 | 25318253 | 25318349 | NR_003318.1 |
| 16798144 | chr15 | 25302006 | 25302102 | NR_003318.1 |
| 16742106 | chr11 | 74165886 | 74178774 | NM_005472.4//KCNE3 |
| 16697893 | chr1 | 2.01E+08 | 201368736 | NM_005558.3//LAD1 |
| 16847095 | chr17 | 56429861 | 56494931 | NM_017763.4//RNF43 |
| 16702547 | chr10 | 13141449 | 13180291 | ENST00000263036//OPTN |
| 16831383 | chr17 | 15848231 | 15879210 | NM_000676.2//ADORA2B |
| 16878583 | chr2 | 29136528 | 29136616 | NR_003074.1 |
| 16918296 | chr20 | 30780306 | 30795594 | NM_002657.3//PLAGL2 |
| 16850426 | chr18 | 48162 | 48272 | ENST00000408514//AP001005.1 |
| 16797375 | chr14 | 1.06E+08 | 106188274 | ENST00000558023//AL901608.1 |
| 16815259 | chr16 | 2961980 | 3001209 | NM_032296.2//FLYWCH1 |
| 17110495 | chrX | 47511191 | 47518579 | NM_004182.3//UXT |
| 16992314 | chr5 | 1.71E+08 | 170710717 | ENST00000516578//snoU13 |
| 17122322 | TCONS_l2_00014091 | 1 | 229 | TCONS_l2_00014091 |
| 16887589 | chr2 | 1.72E+08 | 172414643 | ENST00000409484//CYBRD1 |
| 16935775 | chr22 | 43562628 | 43583139 | NM_015140.3//TTLL12 |
| 17118724 | tc5006985 | 1 | 85 | NR_003939.1 |
| 16988047 | chr5 | 1.12E+08 | 112114166 | ENST00000391068//U6 |
| 16940108 | chr3 | 45633747 | 45633819 | ENST00000516118//AC099539.1 |
| 17109109 | chrX | 12166437 | 12166520 | ENST00000516302//AC002981.1 |
| 16879854 | chr2 | 47294961 | 47298343 | ENST00000421759//AC073283.7.1 |
| 16963646 | chr3 | 1.98E+08 | 197846878 | ENST00000408525//AC073135.1 |
| 16906654 | chr2 | 1.97E+08 | 196535198 | ENST00000364943//U6 |
| 16798152 | chr15 | 25315578 | 25315674 | NR_003323.1 |
| 16897834 | chr2 | 56093097 | 56151298 | NM_001039348.2//EFEMP1 |
| 16949212 | chr3 | 1.85E+08 | 185160107 | ENST00000459417//snoU13 |
| 16957884 | chr3 | 1.2E+08 | 120170100 | NM_007085.4//FSTL1 |
| 16858092 | chr19 | 10216899 | 10226065 | ENST00000393793//PPAN |
| 16732028 | chr11 | 1.19E+08 | 118620973 | ENST00000526274//AP002954.4.1/ |
| 16854145 | chr18 | 18529701 | 18691812 | NM_005406.2//ROCK1 |
| 16894894 | chr2 | 20232411 | 20251789 | NM_014713.4//LAPTM4A |
| 17081071 | chr8 | 1.29E+08 | 129022857 | ENST00000410569//U4 |
| 16967187 | chr4 | 60698893 | 60698994 | ENST00000364806//Y_RNA |
| 17022362 | chr6 | 1.09E+08 | 109416022 | NM_014454.2//SESN1 |
| 16958953 | chr3 | 1.29E+08 | 128902810 | NM_003418.4//CNBP |
| 16889627 | chr2 | 2.03E+08 | 203141241 | NR_003058.1 |
| 16798148 | chr15 | 25310172 | 25310269 | NR_003317.1 |
| 16697395 | chr1 | 1.9E+08 | 189635386 | ENST00000516744//RN5S73 |
| 16884030 | chr2 | 1.09E+08 | 108926371 | NM_001056.3//SULT1C2 |
| 17115884 | chrY | 1470355 | 1484314 | NR_026710.1 |
| 17118262 | chr7 | 66369107 | 66370643 | XR_133450.1 |
| 16817647 | chr16 | 29802041 | 29816706 | ENST00000400751//KIF22 |
| 16850069 | chr17 | 79993757 | 79995573 | NM_016286.3//DCXR |
| 16881838 | chr2 | 75059782 | 75120486 | NM_000189.4//HK2 |
| 16660863 | chr1 | 25071760 | 25170815 | NM_013943.2//CLIC4 |
| 17094468 | chr9 | 67784857 | 67786908 | TCONS_l2_00029970 |
