# Supplementary material for: Building personalized treatment plans for early-stage colorectal cancer patients
Source: Oncotarget. 2017 Jan 13;8(8):13805–17. doi: 10.18632/oncotarget.14638 (PMC5355140; doi:10.18632/oncotarget.14638)
Supplement: Supplementary file 3 [file oncotarget-08-13805-s003.docx]

**Supplementary Table 4 Prediction Score**

| **Recurrence Prediction** | **Score** |
| --- | --- |
| Recurrent1 | -4.3603 |
| Recurrent2 | -4.20277 |
| Recurrent3 | -3.76499 |
| Recurrent4 | -3.64658 |
| Recurrent5 | -3.3142 |
| Recurrent6 | -1.93348 |
| Recurrent7 | -1.58315 |
| Recurrent8 | -1.5456 |
| Recurrent9 | -1.33465 |
| Recurrent10 | -1.00104 |
| Recurrent11 | -0.95299 |
| Recurrent12 | -0.65687 |
| Recurrent13 | -0.56572 |
| Recurrent14 | -0.53994 |
| Recurrent15 | -0.36725 |
| Recurrent16 | -0.31504 |
| Recurrent17 | -0.31343 |
| Recurrent18 | -0.27395 |
| Recurrent19 | -0.25217 |
| Recurrent20 | 0.00106 |
| Recurrent21 | 0.060986 |
| Recurrent22 | 0.068333 |
| Recurrent23 | 0.076382 |
| Recurrent24 | 0.07653 |
| Recurrent25 | 0.077493 |
| Recurrent26 | 0.08527 |
| Recurrent27 | 0.243712 |
| Recurrent28 | 0.288429 |
| Recurrent29 | 0.394023 |
| Recurrent30 | 0.411832 |
| Recurrent31 | 0.522516 |
| Recurrent32 | 0.612554 |
| Recurrent33 | 0.617482 |
| Recurrent34 | 0.646355 |
| Recurrent35 | 0.681412 |
| Recurrent36 | 0.690623 |
| Recurrent37 | 0.691911 |
| Recurrent38 | 0.734904 |
| Recurrent39 | 0.745911 |
| Recurrent40 | 0.750767 |
| Recurrent41 | 0.772083 |
| Recurrent42 | 0.882241 |
| Recurrent43 | 0.899903 |
| Recurrent44 | 0.955316 |
| Recurrent45 | 0.955762 |
| Recurrent46 | 0.960232 |
| Recurrent47 | 0.99755 |
| Recurrent48 | 1.013484 |
| Recurrent49 | 1.103204 |
| Recurrent50 | 1.159736 |
| Recurrent51 | 1.179761 |
| Recurrent52 | 1.255766 |
| Recurrent53 | 1.319589 |
| Recurrent54 | 1.325429 |
| Recurrent55 | 1.365721 |
| Recurrent56 | 1.534269 |
| Recurrent57 | 1.622949 |
| Recurrent58 | 1.623002 |
| Recurrent59 | 1.640896 |
| Recurrent60 | 1.660232 |
| Recurrent61 | 1.688155 |
| Recurrent62 | 1.691625 |
| Recurrent63 | 1.767288 |
| Recurrent64 | 1.809674 |
| Recurrent65 | 1.823293 |
| Recurrent66 | 1.848231 |
| Recurrent67 | 1.862077 |
| Recurrent68 | 1.885636 |
| Recurrent69 | 1.895406 |
| Recurrent70 | 1.951337 |
| Recurrent71 | 1.974117 |
| Recurrent72 | 1.985934 |
| Recurrent73 | 2.144359 |
| Recurrent74 | 2.208705 |
| Recurrent75 | 2.217976 |
| Recurrent76 | 2.223192 |
| Recurrent77 | 2.338517 |
| Recurrent78 | 2.368487 |
| Recurrent79 | 2.379339 |
| Recurrent80 | 2.490085 |
| Recurrent81 | 2.531525 |
| Recurrent82 | 2.653438 |
| Recurrent83 | 2.899544 |
| Recurrent84 | 3.003517 |
| Recurrent85 | 3.357041 |
| Recurrent86 | 3.373689 |
| Recurrent87 | 3.388976 |
| Recurrent88 | 3.449317 |
| Recurrent89 | 3.475516 |
| Recurrent90 | 3.926693 |
| Recurrent91 | 3.994897 |
| Recurrent92 | 4.112926 |
| Recurrent93 | 4.16461 |
| Recurrent94 | 4.457833 |
| Recurrent95 | 5.063617 |
| Recurrent96 | 5.288902 |
| Recurrent97 | 6.143681 |
| NonRecurrent1 | 3.770304 |
| NonRecurrent2 | 3.068029 |
| NonRecurrent3 | 2.438787 |
| NonRecurrent4 | 2.363539 |
| NonRecurrent5 | 2.175008 |
| NonRecurrent6 | 2.073513 |
| NonRecurrent7 | 1.937878 |
| NonRecurrent8 | 1.921732 |
| NonRecurrent9 | 1.898357 |
| NonRecurrent10 | 1.896419 |
| NonRecurrent11 | 1.64662 |
| NonRecurrent12 | 1.625965 |
| NonRecurrent13 | 1.583271 |
| NonRecurrent14 | 1.56267 |
| NonRecurrent15 | 1.466067 |
| NonRecurrent16 | 1.434211 |
| NonRecurrent17 | 1.399484 |
| NonRecurrent18 | 1.22677 |
| NonRecurrent19 | 1.214865 |
| NonRecurrent20 | 1.045528 |
| NonRecurrent21 | 0.973877 |
| NonRecurrent22 | 0.91829 |
| NonRecurrent23 | 0.85158 |
| NonRecurrent24 | 0.820707 |
| NonRecurrent25 | 0.810793 |
| NonRecurrent26 | 0.795408 |
| NonRecurrent27 | 0.777252 |
| NonRecurrent28 | 0.765342 |
| NonRecurrent29 | 0.755688 |
| NonRecurrent30 | 0.753653 |
| NonRecurrent31 | 0.707976 |
| NonRecurrent32 | 0.622696 |
| NonRecurrent33 | 0.619552 |
| NonRecurrent34 | 0.46694 |
| NonRecurrent35 | 0.457889 |
| NonRecurrent36 | 0.413632 |
| NonRecurrent37 | 0.219577 |
| NonRecurrent38 | 0.199915 |
| NonRecurrent39 | 0.171664 |
| NonRecurrent40 | 0.13009 |
| NonRecurrent41 | 0.126701 |
| NonRecurrent42 | 0.033611 |
| NonRecurrent43 | 0.022783 |
| NonRecurrent44 | 0.019424 |
| NonRecurrent45 | -0.01372 |
| NonRecurrent46 | -0.0429 |
| NonRecurrent47 | -0.06963 |
| NonRecurrent48 | -0.21779 |
| NonRecurrent49 | -0.25058 |
| NonRecurrent50 | -0.28683 |
| NonRecurrent51 | -0.28812 |
| NonRecurrent52 | -0.30186 |
| NonRecurrent53 | -0.31562 |
| NonRecurrent54 | -0.34487 |
| NonRecurrent55 | -0.41115 |
| NonRecurrent56 | -0.43385 |
| NonRecurrent57 | -0.44141 |
| NonRecurrent58 | -0.49615 |
| NonRecurrent59 | -0.53272 |
| NonRecurrent60 | -0.55214 |
| NonRecurrent61 | -0.57701 |
| NonRecurrent62 | -0.57924 |
| NonRecurrent63 | -0.65603 |
| NonRecurrent64 | -0.68972 |
| NonRecurrent65 | -0.81504 |
| NonRecurrent66 | -0.84731 |
| NonRecurrent67 | -0.85591 |
| NonRecurrent68 | -0.85823 |
| NonRecurrent69 | -0.92297 |
| NonRecurrent70 | -0.93967 |
| NonRecurrent71 | -0.95124 |
| NonRecurrent72 | -0.97721 |
| NonRecurrent73 | -1.01198 |
| NonRecurrent74 | -1.08035 |
| NonRecurrent75 | -1.08047 |
| NonRecurrent76 | -1.092 |
| NonRecurrent77 | -1.13871 |
| NonRecurrent78 | -1.19648 |
| NonRecurrent79 | -1.2037 |
| NonRecurrent80 | -1.27592 |
| NonRecurrent81 | -1.38032 |
| NonRecurrent82 | -1.39924 |
| NonRecurrent83 | -1.47103 |
| NonRecurrent84 | -1.51373 |
| NonRecurrent85 | -1.52346 |
| NonRecurrent86 | -1.6215 |
| NonRecurrent87 | -1.64 |
| NonRecurrent88 | -1.79262 |
| NonRecurrent89 | -1.80499 |
| NonRecurrent90 | -1.83487 |
| NonRecurrent91 | -1.85649 |
| NonRecurrent92 | -1.87762 |
| NonRecurrent93 | -1.93077 |
| NonRecurrent94 | -1.95679 |
| NonRecurrent95 | -1.96455 |
| NonRecurrent96 | -2.0521 |
| NonRecurrent97 | -2.13346 |
| NonRecurrent98 | -2.24561 |
| NonRecurrent99 | -2.26435 |
| NonRecurrent100 | -2.27098 |
| NonRecurrent101 | -2.28566 |
| NonRecurrent102 | -2.29145 |
| NonRecurrent103 | -2.3545 |
| NonRecurrent104 | -2.39778 |
| NonRecurrent105 | -2.46186 |
| NonRecurrent106 | -2.50523 |
| NonRecurrent107 | -2.50554 |
| NonRecurrent108 | -2.50627 |
| NonRecurrent109 | -2.70895 |
| NonRecurrent110 | -2.72544 |
| NonRecurrent111 | -2.75263 |
| NonRecurrent112 | -2.81044 |
| NonRecurrent113 | -2.82103 |
| NonRecurrent114 | -2.85528 |
| NonRecurrent115 | -2.90947 |
| NonRecurrent116 | -2.92851 |
| NonRecurrent117 | -2.97954 |
| NonRecurrent118 | -3.01078 |
| NonRecurrent119 | -3.02404 |
| NonRecurrent120 | -3.03183 |
| NonRecurrent121 | -3.03676 |
| NonRecurrent122 | -3.25832 |
| NonRecurrent123 | -3.29474 |
| NonRecurrent124 | -3.30335 |
| NonRecurrent125 | -3.33751 |
| NonRecurrent126 | -3.43428 |
| NonRecurrent127 | -3.44445 |
| NonRecurrent128 | -3.4929 |
| NonRecurrent129 | -3.49425 |
| NonRecurrent130 | -3.66174 |
| NonRecurrent131 | -3.70325 |
| NonRecurrent132 | -3.93847 |
| NonRecurrent133 | -4.14674 |
| NonRecurrent134 | -4.40306 |
| NonRecurrent135 | -4.67335 |
| NonRecurrent136 | -4.83998 |
| NonRecurrent137 | -5.14114 |
| NonRecurrent138 | -5.44007 |

| **FOLFOX Prediction** | **Score** |
| --- | --- |
| Non-responder1 | -5.689929008 |
| Non-responder2 | -5.257318497 |
| Non-responder3 | -4.942192554 |
| Non-responder4 | -4.731384277 |
| Non-responder5 | -4.59233427 |
| Non-responder6 | -4.389923334 |
| Non-responder7 | -4.229794502 |
| Non-responder8 | -4.059282541 |
| Non-responder9 | -3.665863037 |
| Non-responder10 | -3.353738308 |
| Non-responder11 | -2.787751198 |
| Non-responder12 | -2.506281137 |
| Non-responder13 | -2.377666473 |
| Non-responder14 | -2.375307918 |
| Non-responder15 | -2.218203425 |
| Non-responder16 | -2.198395967 |
| Non-responder17 | -2.134885669 |
| Non-responder18 | -2.023835063 |
| Non-responder19 | -2.021446943 |
| Non-responder20 | -1.74378264 |
| Non-responder21 | -1.551010966 |
| Non-responder22 | -1.190933883 |
| Non-responder23 | -1.167894721 |
| Non-responder24 | -1.122944832 |
| Non-responder25 | -1.093041003 |
| Non-responder26 | -1.053685784 |
| Non-responder27 | -1.033155382 |
| Non-responder28 | -0.794781744 |
| Non-responder29 | -0.71272552 |
| Non-responder30 | -0.45700166 |
| Non-responder31 | -0.447385874 |
| Non-responder32 | -0.307613149 |
| Non-responder33 | -0.222464383 |
| Non-responder34 | -0.085664034 |
| Non-responder35 | 0.006422937 |
| Non-responder36 | 0.033998311 |
| Non-responder37 | 0.035376728 |
| Non-responder38 | 0.068328857 |
| Non-responder39 | 0.105296612 |
| Non-responder40 | 0.206585586 |
| Non-responder41 | 0.240651131 |
| Non-responder42 | 0.264231384 |
| Non-responder43 | 0.326342344 |
| Non-responder44 | 0.326728284 |
| Non-responder45 | 0.412664771 |
| Non-responder46 | 0.43315053 |
| Non-responder47 | 0.58487916 |
| Non-responder48 | 0.660811663 |
| Non-responder49 | 0.679080844 |
| Non-responder50 | 0.757704258 |
| Non-responder51 | 0.775345087 |
| Non-responder52 | 0.794976354 |
| Non-responder53 | 0.901688337 |
| Non-responder54 | 0.979800701 |
| Non-responder55 | 0.985687256 |
| Non-responder56 | 0.99202323 |
| Non-responder57 | 1.16415143 |
| Non-responder58 | 1.251966715 |
| Non-responder59 | 1.320524693 |
| Non-responder60 | 1.381970167 |
| Non-responder61 | 1.391613483 |
| Non-responder62 | 1.396687508 |
| Non-responder63 | 1.426107764 |
| Non-responder64 | 1.835962296 |
| Responder1 | 1.140373707 |
| Responder2 | 0.944082022 |
| Responder3 | 0.86907053 |
| Responder4 | 0.759449005 |
| Responder5 | 0.720926166 |
| Responder6 | 0.579230666 |
| Responder7 | 0.527842999 |
| Responder8 | 0.245984674 |
| Responder9 | 0.204805434 |
| Responder10 | 0.182543039 |
| Responder11 | 0.145724475 |
| Responder12 | 0.108310878 |
| Responder13 | -0.064519674 |
| Responder14 | -0.078847647 |
| Responder15 | -0.154736131 |
| Responder16 | -0.162542462 |
| Responder17 | -0.248505533 |
| Responder18 | -0.256485701 |
| Responder19 | -0.272609204 |
| Responder20 | -0.318008155 |
| Responder21 | -0.366887003 |
| Responder22 | -0.373982519 |
| Responder23 | -0.522980832 |
| Responder24 | -0.609617658 |
| Responder25 | -0.616314873 |
| Responder26 | -0.630083293 |
| Responder27 | -0.66582942 |
| Responder28 | -0.666047573 |
| Responder29 | -0.690690562 |
| Responder30 | -0.786906123 |
| Responder31 | -0.79495427 |
| Responder32 | -0.837122709 |
| Responder33 | -0.846614987 |
| Responder34 | -0.870549411 |
| Responder35 | -0.924574614 |
| Responder36 | -0.944312334 |
| Responder37 | -0.949657172 |
| Responder38 | -0.973232538 |
| Responder39 | -0.995571792 |
| Responder40 | -1.005791843 |
| Responder41 | -1.013750076 |
| Responder42 | -1.146200418 |
| Responder43 | -1.1836285 |
| Responder44 | -1.234758675 |
| Responder45 | -1.373777986 |
| Responder46 | -1.425862551 |
| Responder47 | -1.443466365 |
| Responder48 | -1.456345141 |
| Responder49 | -1.520458341 |
| Responder50 | -1.535327435 |
| Responder51 | -1.573006988 |
| Responder52 | -1.583978772 |
| Responder53 | -1.696345329 |
| Responder54 | -1.71580112 |
| Responder55 | -1.757418394 |
| Responder56 | -1.806540251 |
| Responder57 | -1.831594706 |
| Responder58 | -1.929350376 |
| Responder59 | -2.092460632 |
| Responder60 | -2.103808641 |
| Responder61 | -2.110239029 |
| Responder62 | -2.204056382 |
| Responder63 | -2.25436759 |
| Responder64 | -2.290179014 |
| Responder65 | -2.300149918 |
| Responder66 | -2.401645899 |
| Responder67 | -2.483119845 |
| Responder68 | -2.708972692 |
| Responder69 | -2.781228065 |
| Responder70 | -3.028367281 |
| Responder71 | -3.058828115 |
| Responder72 | -3.071271896 |
| Responder73 | -3.466289997 |
| Responder74 | -3.471319914 |
| Responder75 | -3.675273418 |
| Responder76 | -3.713211775 |
| Responder77 | -3.722907305 |
| Responder78 | -3.726540565 |
| Responder79 | -3.746594667 |
| Responder80 | -3.804193497 |
| Responder81 | -4.044823647 |
| Responder82 | -4.071738482 |
| Responder83 | -4.146587372 |
| Responder84 | -4.426373959 |
| Responder85 | -4.446940899 |
| Responder86 | -4.573089123 |
| Responder87 | -4.574866295 |
| Responder88 | -4.619122505 |
| Responder89 | -4.705394268 |
| Responder90 | -4.743422985 |
| Responder91 | -4.745420933 |
| Responder92 | -4.872329235 |
| Responder93 | -4.924072742 |
| Responder94 | -5.036393166 |
| Responder95 | -5.185083866 |
| Responder96 | -5.260191441 |
| Responder97 | -5.440135002 |
| Responder98 | -5.518752098 |
| Responder99 | -5.521591187 |
| Responder100 | -5.656777382 |
| Responder101 | -6.04826498 |
| Responder102 | -6.175846577 |

| 5FU Prediction | Score |
| --- | --- |
| Non-responder1 | -3.66744 |
| Non-responder2 | -2.87699 |
| Non-responder3 | -2.70322 |
| Non-responder4 | -2.2019 |
| Non-responder5 | -1.94011 |
| Non-responder6 | -1.69807 |
| Non-responder7 | -1.67794 |
| Non-responder8 | -1.66861 |
| Non-responder9 | -1.58601 |
| Non-responder10 | -1.57246 |
| Non-responder11 | -1.52509 |
| Non-responder12 | -1.49155 |
| Non-responder13 | -1.44338 |
| Non-responder14 | -1.19172 |
| Non-responder15 | -1.18461 |
| Non-responder16 | -1.16859 |
| Non-responder17 | -1.0541 |
| Non-responder18 | -0.93259 |
| Non-responder19 | -0.88844 |
| Non-responder20 | -0.86468 |
| Non-responder21 | -0.78203 |
| Non-responder22 | -0.7176 |
| Non-responder23 | -0.55694 |
| Non-responder24 | -0.55026 |
| Non-responder25 | -0.47794 |
| Non-responder26 | -0.36681 |
| Non-responder27 | -0.31201 |
| Non-responder28 | -0.10242 |
| Non-responder29 | 0.066269 |
| Non-responder30 | 0.155888 |
| Non-responder31 | 0.262509 |
| Non-responder32 | 0.505156 |
| Non-responder33 | 0.618895 |
| Non-responder34 | 0.64282 |
| Non-responder35 | 0.662557 |
| Non-responder36 | 0.820571 |
| Non-responder37 | 0.833441 |
| Non-responder38 | 0.852971 |
| Non-responder39 | 0.88336 |
| Non-responder40 | 1.052365 |
| Non-responder41 | 1.093818 |
| Non-responder42 | 1.174313 |
| Non-responder43 | 1.196132 |
| Non-responder44 | 1.280284 |
| Non-responder45 | 1.350544 |
| Non-responder46 | 1.451444 |
| Non-responder47 | 1.531623 |
| Non-responder48 | 1.63528 |
| Non-responder49 | 1.731827 |
| Non-responder50 | 1.772386 |
| Non-responder51 | 1.964253 |
| Non-responder52 | 1.987118 |
| Non-responder53 | 1.992252 |
| Non-responder54 | 2.027676 |
| Non-responder55 | 2.040965 |
| Non-responder56 | 2.093949 |
| Non-responder57 | 2.156574 |
| Non-responder58 | 2.170927 |
| Non-responder59 | 2.259577 |
| Non-responder60 | 2.268444 |
| Non-responder61 | 2.288517 |
| Non-responder62 | 2.331486 |
| Non-responder63 | 2.35533 |
| Non-responder64 | 2.373106 |
| Non-responder65 | 2.383723 |
| Non-responder66 | 2.419031 |
| Non-responder67 | 2.460244 |
| Non-responder68 | 2.703436 |
| Non-responder69 | 2.76166 |
| Non-responder70 | 2.803349 |
| Non-responder71 | 2.916661 |
| Non-responder72 | 3.115098 |
| Non-responder73 | 3.460814 |
| Responder1 | 2.258128 |
| Responder2 | 1.593968 |
| Responder3 | 1.589314 |
| Responder4 | 1.510668 |
| Responder5 | 1.494432 |
| Responder6 | 1.287725 |
| Responder7 | 1.281436 |
| Responder8 | 1.213545 |
| Responder9 | 1.196437 |
| Responder10 | 1.190374 |
| Responder11 | 1.124631 |
| Responder12 | 0.91692 |
| Responder13 | 0.879459 |
| Responder14 | 0.856088 |
| Responder15 | 0.796208 |
| Responder16 | 0.772386 |
| Responder17 | 0.674821 |
| Responder18 | 0.662059 |
| Responder19 | 0.384682 |
| Responder20 | 0.302319 |
| Responder21 | 0.290815 |
| Responder22 | 0.284043 |
| Responder23 | 0.268829 |
| Responder24 | 0.09496 |
| Responder25 | 0.09172 |
| Responder26 | 0.065879 |
| Responder27 | 0.061451 |
| Responder28 | 0.031531 |
| Responder29 | 0.022434 |
| Responder30 | 0.000542 |
| Responder31 | -0.00684 |
| Responder32 | -0.03001 |
| Responder33 | -0.12324 |
| Responder34 | -0.12985 |
| Responder35 | -0.16243 |
| Responder36 | -0.17925 |
| Responder37 | -0.18545 |
| Responder38 | -0.23282 |
| Responder39 | -0.2533 |
| Responder40 | -0.27673 |
| Responder41 | -0.31729 |
| Responder42 | -0.46341 |
| Responder43 | -0.51407 |
| Responder44 | -0.56945 |
| Responder45 | -0.5824 |
| Responder46 | -0.58283 |
| Responder47 | -0.58506 |
| Responder48 | -0.69679 |
| Responder49 | -0.70317 |
| Responder50 | -0.70826 |
| Responder51 | -0.7485 |
| Responder52 | -0.77503 |
| Responder53 | -0.7988 |
| Responder54 | -0.83924 |
| Responder55 | -0.88122 |
| Responder56 | -0.94862 |
| Responder57 | -0.96766 |
| Responder58 | -1.00732 |
| Responder59 | -1.03032 |
| Responder60 | -1.06128 |
| Responder61 | -1.10599 |
| Responder62 | -1.13064 |
| Responder63 | -1.20293 |
| Responder64 | -1.27248 |
| Responder65 | -1.27667 |
| Responder66 | -1.29159 |
| Responder67 | -1.32549 |
| Responder68 | -1.39179 |
| Responder69 | -1.44941 |
| Responder70 | -1.51161 |
| Responder71 | -1.53428 |
| Responder72 | -1.56033 |
| Responder73 | -1.57582 |
| Responder74 | -1.63368 |
| Responder75 | -1.66404 |
| Responder76 | -1.71735 |
| Responder77 | -1.73296 |
| Responder78 | -1.737 |
| Responder79 | -1.76318 |
| Responder80 | -1.85876 |
| Responder81 | -1.90438 |
| Responder82 | -1.92955 |
| Responder83 | -1.93687 |
| Responder84 | -1.94038 |
| Responder85 | -2.03795 |
| Responder86 | -2.06929 |
| Responder87 | -2.12604 |
| Responder88 | -2.19333 |
| Responder89 | -2.20832 |
| Responder90 | -2.22789 |
| Responder91 | -2.23982 |
| Responder92 | -2.26866 |
| Responder93 | -2.35762 |
| Responder94 | -2.47927 |
| Responder95 | -2.49904 |
| Responder96 | -2.54407 |
| Responder97 | -2.56074 |
| Responder98 | -2.62522 |
| Responder99 | -2.65212 |
| Responder100 | -2.71955 |
| Responder101 | -2.72019 |
| Responder102 | -2.75597 |
| Responder103 | -2.83782 |
| Responder104 | -2.90031 |
| Responder105 | -2.90627 |
| Responder106 | -2.98747 |
| Responder107 | -3.17353 |
| Responder108 | -3.23385 |
| Responder109 | -3.34662 |
| Responder110 | -3.52466 |
| Responder111 | -3.78399 |
| Responder112 | -4.0094 |
| Responder113 | -4.14142 |
| Responder114 | -4.39747 |
| Responder115 | -4.41221 |
| Responder116 | -4.47513 |
| Responder117 | -4.52228 |
| Responder118 | -4.77871 |
| Responder119 | -5.5167 |
